# Supplementary material for: Little evidence for a role of facial mimicry in the transmission of stress from parents to adolescent children
Source: Commun Psychol. 2025 May 16;3:78. doi: 10.1038/s44271-025-00260-3 (PMC12084340; doi:10.1038/s44271-025-00260-3)
Supplement: Supplementary file 2 — Supplementary Information [file 44271_2025_260_MOESM2_ESM.pdf]

Supplementary Information for:

Little evidence for a role of facial mimicry in the transmission of stress from parents to adolescent children.

Jost Ulrich Blasberg[1], Philipp Kanske[2], Veronika Engert[1,3,4]

[1]Institute of Psychosocial Medicine, Psychotherapy and Psychooncology, Jena University Hospital, Friedrich-Schiller University, Jena, Germany

[2] Clinical Psychology and Behavioral Neuroscience, Faculty of Psychology, Technische Universität Dresden, Dresden, Germany

[3]German Center for Mental Health (DZPG), partner site Halle-Jena-Magdeburg, Jena, Germany

[4]Center for Intervention and Research in adaptive and maladaptive brain Circuits underlying mental health (C-I-R-C), Halle-Jena-Magdeburg, Jena, Germany

Supplementary Methods

Table S1. Missing data points per stress marker

Dependent variable	Sample size	Data points imputed (%)
Full sample	77	
Cortisol	70	0.80 %
Heart rate	37	1.51 %
HRV	37	1.61 %
Subjective stress (STAI)	70	2.80 %
Empathic concern	71	1.30 %
Personal distress	69	1.48 %

Figure S1. Scree plot for exploratory factor analysis including all AU sync times.

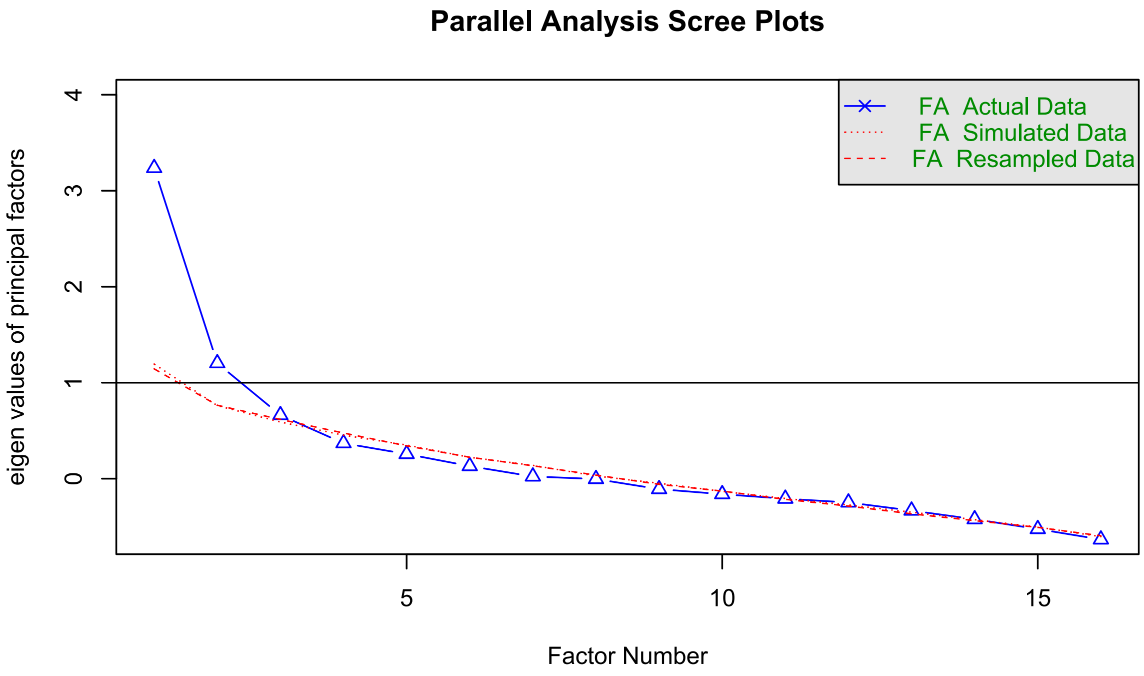

Note. Parallel analysis reveals a two-factor solution for an SEM including the sixteen AU sync times as observed variables.

Figure S2. Correlations between action unit sync times across participants.

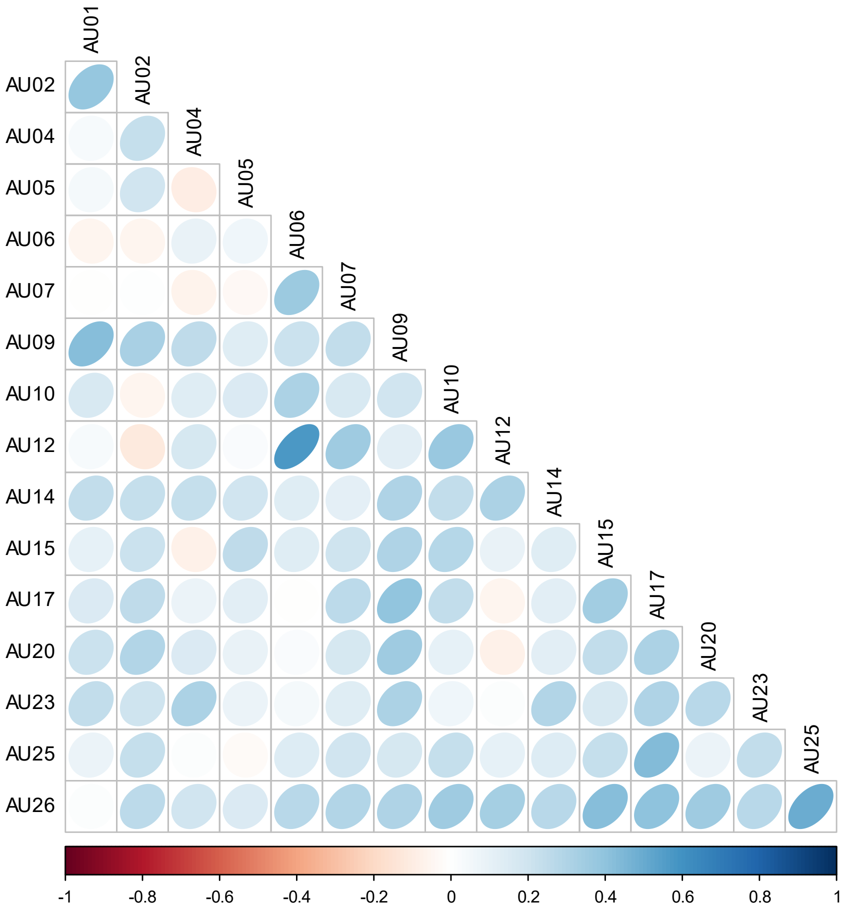

Note. Positive (blue) and negative (red) correlations between action unit sync times.

Table S2. Multiple linear regressions on adolescent cortisol, heart rate, HF-HRV, state empathic concern and state personal distress AUCi in relation to WCLR derived mimicry.

	Adolescent AUCi Cortisol		
Coefficient	*Estimates*	*CI*	*p*
(Intercept)	-18.88	-26.28 – -11.48	<0.001
AUCi cortisol (parent)	-2.13	-10.05 – 5.80	0.594
Mimicry (lf1)	-3.48	-11.63 – 4.67	0.397
Mimicry (lf2)	0.54	-7.61 – 8.69	0.895
AUCi (parent)—Mimicry (lf1)	-0.54	-9.19 – 8.11	0.901
AUCi (parent)—Mimicry (lf2)	7.05	-0.98 – 15.08	0.084
Observations	70		
R^2 / R^2 adjusted	0.070 / -0.003		
AIC	687.149		
	Adolescent AUCi Heart rate		
Coefficient	*Estimates*	*CI*	*p*
(Intercept)	33.38	-130.13 – 196.90	0.680
AUCi heart-rate (parent)	241.69	-15.82 – 499.19	0.065
Mimicry (lf1)	91.09	-95.43 – 277.62	0.327
Mimicry (lf2)	-45.45	-212.42 – 121.51	0.583
AUCi (parent)—Mimicry (lf1)	-109.25	-319.18 – 100.69	0.297
AUCi (parent)—Mimicry (lf2)	137.67	-126.82 – 402.15	0.297
Observations	37		
R^2 / R^2 adjusted	0.121 / -0.021		
AIC	569.118		
	Adolescent AUCi HRV		
Coefficient	*Estimates*	*CI*	*p*
(Intercept)	-0.52	-10.02 – 8.98	0.911
AUCi HRV (parent)	11.83	-3.04 – 26.71	0.115
Mimicry (lf1)	-1.25	-12.15 – 9.65	0.817
Mimicry (lf2)	3.93	-5.57 – 13.42	0.406
AUCi (parent)—Mimicry (lf1)	-14.43	-27.11 – -1.76	0.027
AUCi (parent)—Mimicry (lf2)	-4.90	-22.42 – 12.62	0.572
Observations	37		
R^2 / R^2 adjusted	0.094 / -0.052		
AIC	359.067		
	Adolescent AUCi STAI		
Coefficient	*Estimates*	*CI*	*p*
(Intercept)	115.37	13.27 – 217.47	0.027
AUCi STAI (parent)	36.86	-67.72 – 141.45	0.484
Mimicry (lf1)	103.11	-10.15 – 216.37	0.074
Mimicry (lf2)	-19.71	-132.60 – 93.18	0.728
AUCi (parent)—Mimicry (lf1)	5.48	-158.44 – 169.41	0.947
AUCi (parent)—Mimicry (lf2)	27.64	-78.52 – 133.80	0.605
Observations	70		
R^2 / R^2 adjusted	0.098 / 0.027		
AIC	1051.738		
	Adolescent AUCi Empathic Concern		
Coefficient	*Estimates*	*CI*	*p*

(Intercept)	-59.30	-147.32 – 28.73	0.183
Mimicry (lf1)	32.30	-65.34 – 129.95	0.511
Mimicry (lf2)	-108.55	-206.19 – -10.91	0.030
Observations	71		
R^2 / R^2 adjusted	0.033 / 0.005		
AIC	1049.406		
	Adolescent AUCi Personal Distress		
Coefficient	*Estimates*	*CI*	*p*
(Intercept)	89.86	-24.65 – 204.37	0.122
Mimicry (lf1)	96.44	-29.69 – 222.57	0.132
Mimicry (lf2)	-45.71	-171.50 – 80.08	0.471
Observations	69		
R^2 / R^2 adjusted	0.070 / 0.042		
AIC	1050.600		

Note. AUCi = area under the curve with respect to increase; Mimicry (lf1) = positive latent mimicry factor; Mimicry (lf2) = negative latent mimicry factor; AIC = Akaike Information Criterion

Figure S3. AU occurrence rates in % for parents and adolescents.

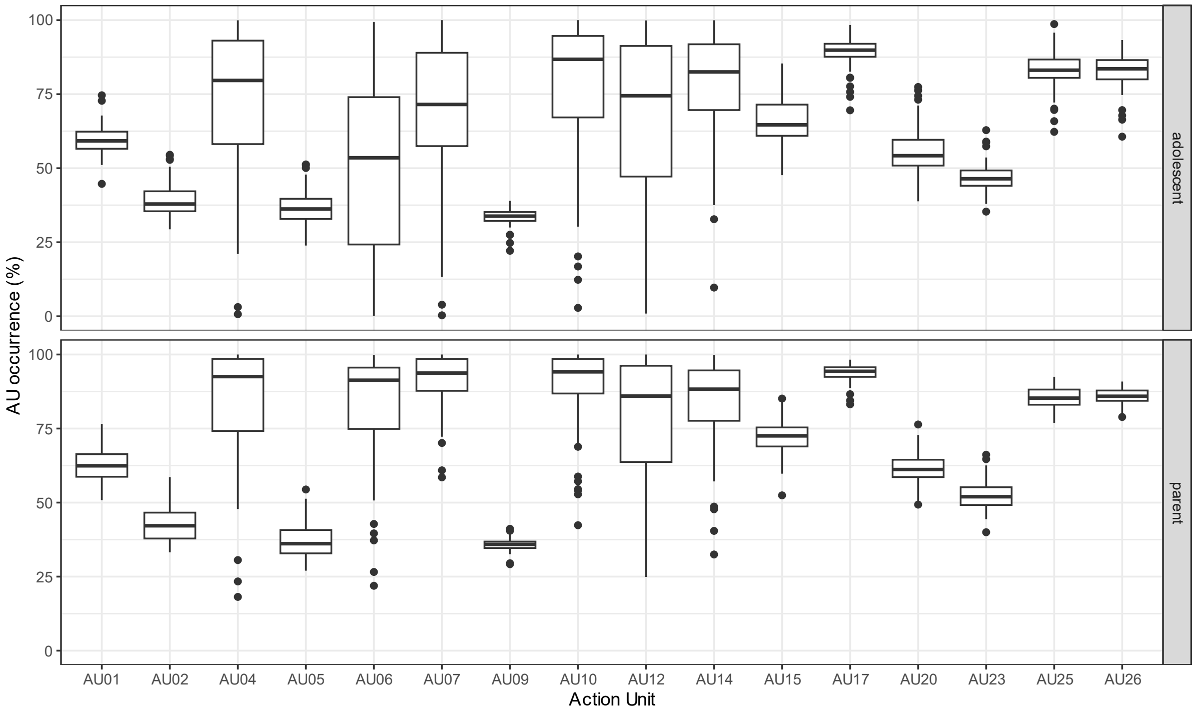

Note. Boxplots with means (horizontal line), standard errors (antenna) and outliers (points).

Figure S4. AU mean intensity for parents and adolescents.

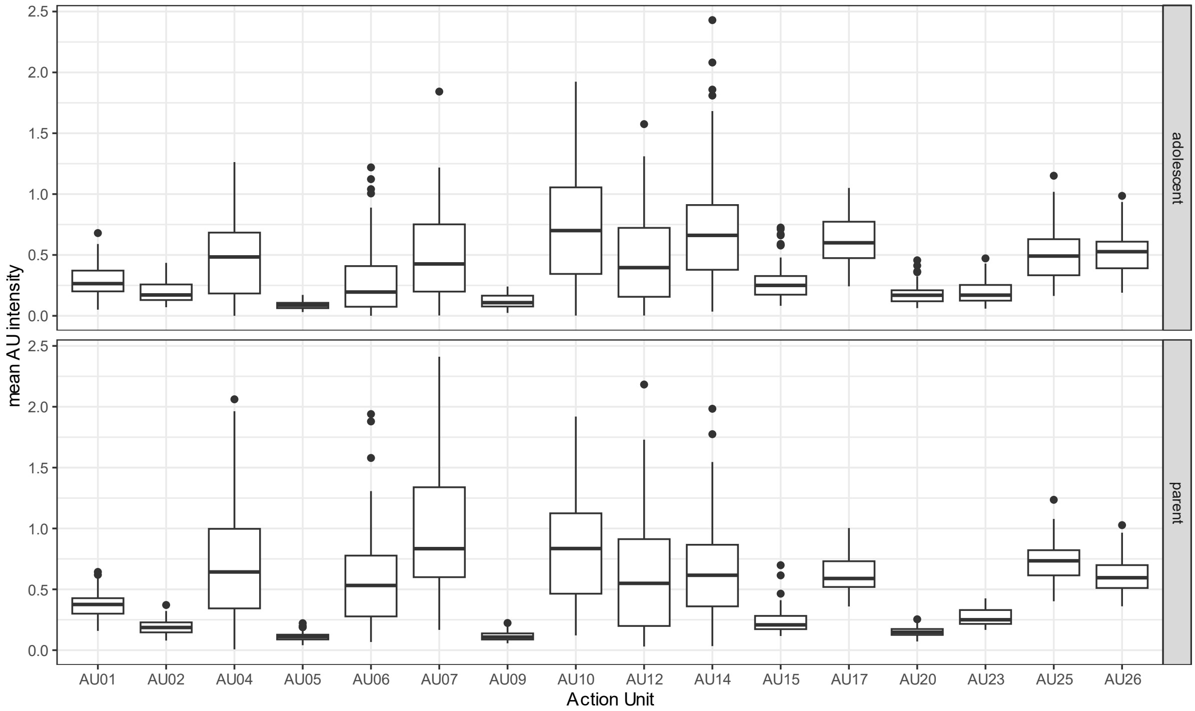

Note. Boxplots with means (horizontal line), standard errors (antenna) and outliers (points).

Figure S5. Mean lag of sync intervals.

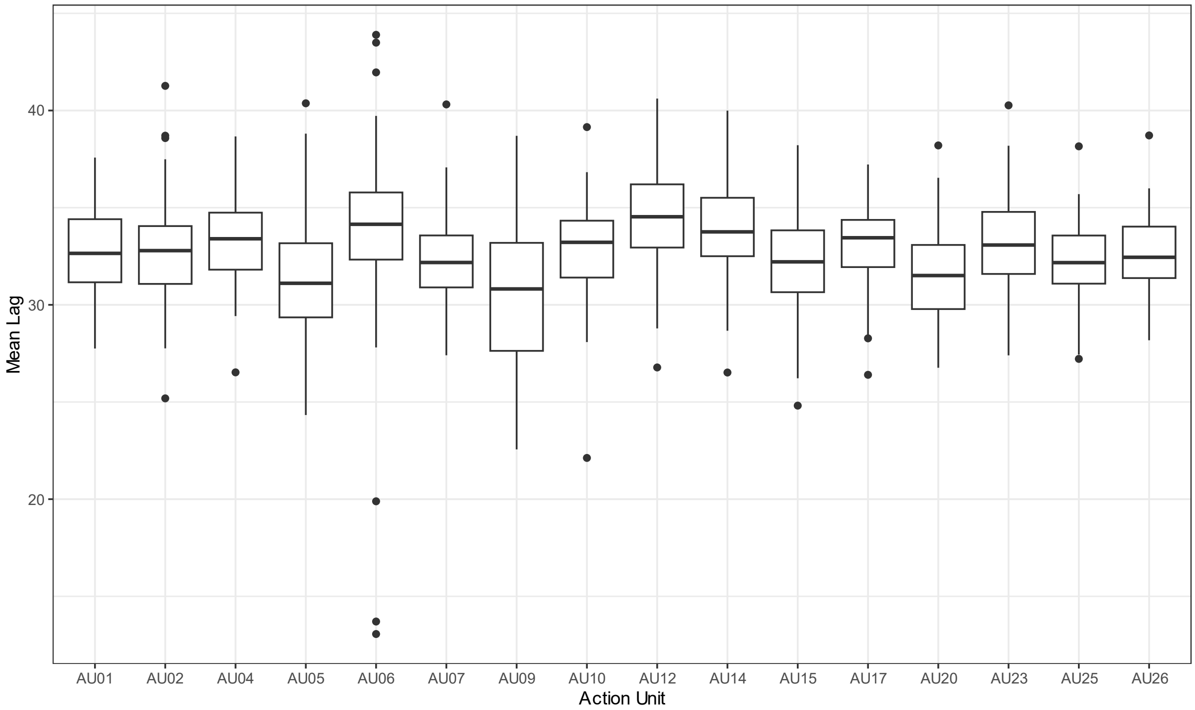

Note. Boxplots with means (horizontal line), standard errors (antenna) and outliers (points).

Figure S6. Mean R^2_{cc} of sync intervals.

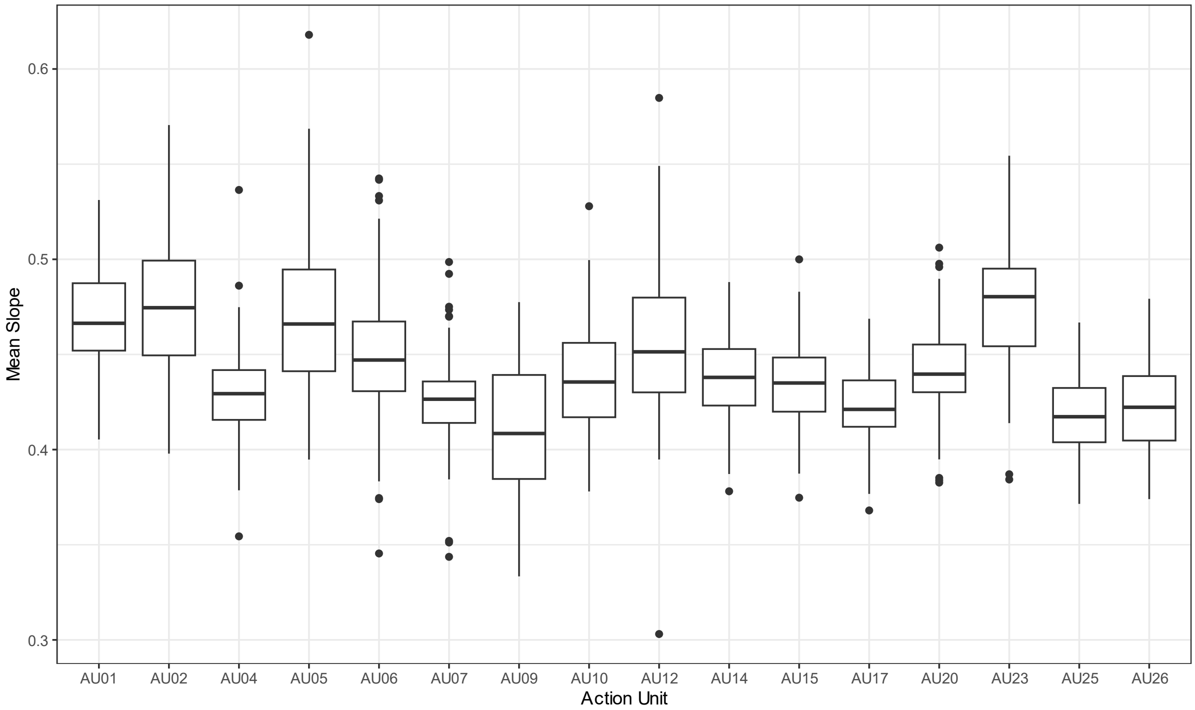

Note. Boxplots with means (horizontal line), standard errors (antenna) and outliers (points).

WCLC sensitivity analysis.

Because we initially preregistered a windowed-cross-lagged-correlation (WCLC) approach and later decided to switch to windowed-cross-lagged-regression (WCLR), in the following the results of a sensitivity analysis using WCLC will be reported.

An exploratory factor analysis of sync times calculated via WCLC resulted in a highly similar factor structure. The only difference was that using WCLR, the first latent factor did not include AU05 sync times (see Figure S9).

Figure S7. SEM factor solution for action unit sync times across participants using WCLC.

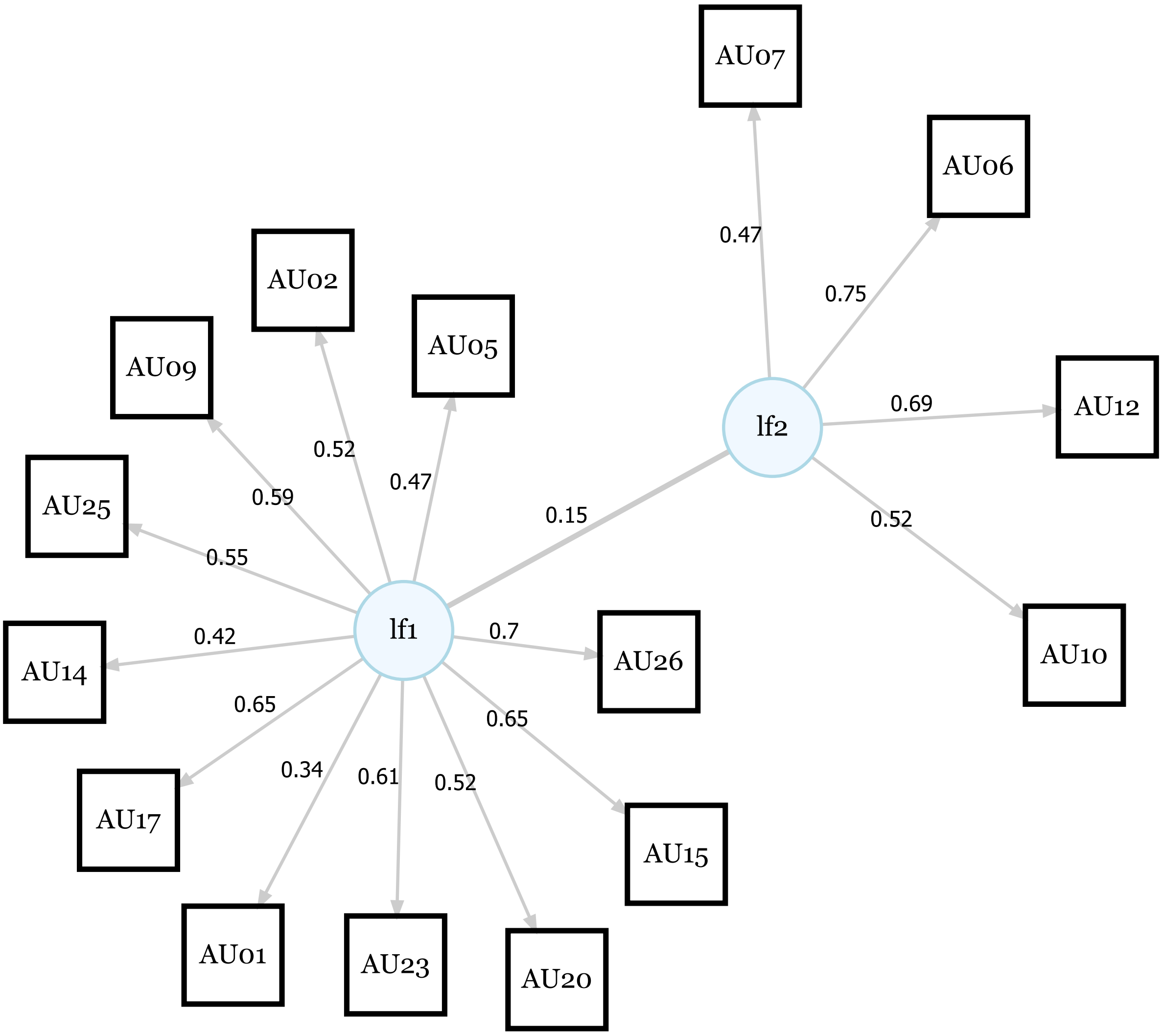

Note. The EFA revealed a two-factor solution. The first latent factor (lf1) loaded onto AU01, AU02, AU05, AU09, AU14, AU15, AU17, AU20, AU23, AU25 and AU26 (various negative emotions). The second latent factor (ldf2) loaded onto AU06, AU07, AU10 and AU12 (smiling or contempt). None of the factors loaded onto AU04 sync times.

Table S3. Multiple linear regressions on adolescent cortisol, heart rate, HF-HRV, state empathic concern and state personal distress AUCi in relation to WCLC derived mimicry

	Adolescent AUCi Cortisol		
Coefficient	*Estimates*	*CI*	*p*
(Intercept)	-19.04	-26.38 – -11.70	<0.001
AUCi cortisol (parent)	-0.93	-8.68 – 6.81	0.810
Mimicry (lf1)	-2.44	-10.12 – 5.25	0.529
Mimicry (lf2)	1.52	-5.93 – 8.98	0.684
AUCi (parent)—Mimicry (lf1)	-6.05	-13.98 – 1.88	0.133
AUCi (parent)—Mimicry (lf2)	7.53	-0.37 – 15.42	0.061
Observations	70		
R^2 / R^2 adjusted	0.070 / -0.003		
AIC	687.149		
	Adolescent AUCi Heart rate		
Coefficient	*Estimates*	*CI*	*p*
(Intercept)	58.47	-98.68 – 215.63	0.454
AUCi heart-rate (parent)	204.43	-9.48 – 418.35	0.060
Mimicry (lf1)	9.25	-149.57 – 168.06	0.906
Mimicry (lf2)	30.94	-117.40 – 179.28	0.674
AUCi (parent)—Mimicry (lf1)	-143.23	-331.24 – 44.78	0.131
AUCi (parent)—Mimicry (lf2)	136.27	-154.75 – 427.29	0.347
Observations	37		
R^2 / R^2 adjusted	0.121 / -0.021		
AIC	569.118		
	Adolescent AUCi HRV		
Coefficient	*Estimates*	*CI*	*p*
(Intercept)	-0.34	-10.29 – 9.61	0.945
AUCi HRV (parent)	7.56	-6.25 – 21.38	0.273
Mimicry (lf1)	-2.77	-12.88 – 7.33	0.580
Mimicry (lf2)	2.90	-5.57 – 11.37	0.490
AUCi (parent)—Mimicry (lf1)	-5.67	-17.60 – 6.26	0.340
AUCi (parent)—Mimicry (lf2)	-4.39	-21.89 – 13.12	0.613
Observations	37		
R^2 / R^2 adjusted	0.094 / -0.052		
AIC	359.067		
	Adolescent AUCi STAI		
Coefficient	*Estimates*	*CI*	*p*
(Intercept)	110.16	11.98 – 208.34	0.028
AUCi STAI (parent)	35.75	-66.61 – 138.11	0.488
Mimicry (lf1)	126.25	25.37 – 227.13	0.015
Mimicry (lf2)	44.87	-56.82 – 146.56	0.381
AUCi (parent)—Mimicry (lf1)	10.69	-128.57 – 149.95	0.879
AUCi (parent)—Mimicry (lf2)	37.57	-44.98 – 120.12	0.367
Observations	70		
R^2 / R^2 adjusted	0.098 / 0.027		
AIC	1051.738		
	Adolescent AUCi Empathic Concern		
Coefficient	*Estimates*	*CI*	*p*

(Intercept)	-66.11	-154.73 – 22.51	0.141
Mimicry (lf1)	45.55	-45.22 – 136.32	0.320
Mimicry (lf2)	-81.30	-172.07 – 9.48	0.078
Observations	71		
R^2 / R^2 adjusted	0.033 / 0.005		
AIC	1049.406		
	Adolescent AUCi Personal Distress		
Coefficient	*Estimates*	*CI*	*p*
(Intercept)	81.62	-32.79 – 196.02	0.159
Mimicry (lf1)	81.17	-34.39 – 196.74	0.166
Mimicry (lf2)	-16.71	-133.54 – 100.11	0.776
Observations	69		
R^2 / R^2 adjusted	0.070 / 0.042		
AIC	1050.600		

Note. AUCi = area under the curve with respect to increase; Mimicry (lf1) = positive latent mimicry factor; Mimicry (lf2) = negative latent mimicry factor; AIC = Akaike Information Criterion.
