# Supplementary material for: Little evidence for a role of facial mimicry in the transmission of stress from parents to adolescent children
Source: Commun Psychol. 2025 May 16;3:78. doi: 10.1038/s44271-025-00260-3 (PMC12084340; doi:10.1038/s44271-025-00260-3)
Supplement: Supplementary file 3 — Reporting Summary [file 44271_2025_260_MOESM3_ESM.pdf]

nature portfolio

Corresponding author(s): *Double-blind peer review submissions: write DBPR and your manuscript number here instead of author names.*

Last updated by author(s): *YYYY-MM-DD*

Reporting Summary

Nature Portfolio wishes to improve the reproducibility of the work that we publish. This form provides structure for consistency and transparency in reporting. For further information on Nature Portfolio policies, see our Editorial Policies and the Editorial Policy Checklist.

Statistics

For all statistical analyses, confirm that the following items are present in the figure legend, table legend, main text, or Methods section.

n/a	Confirmed	
☐	☒	The exact sample size (*n*) for each experimental group/condition, given as a discrete number and unit of measurement
☐	☒	A statement on whether measurements were taken from distinct samples or whether the same sample was measured repeatedly
☐	☒	The statistical test(s) used AND whether they are one- or two-sided<br>*Only common tests should be described solely by name; describe more complex techniques in the Methods section.*
☐	☒	A description of all covariates tested
☐	☒	A description of any assumptions or corrections, such as tests of normality and adjustment for multiple comparisons
☐	☒	A full description of the statistical parameters including central tendency (e.g. means) or other basic estimates (e.g. regression coefficient) AND variation (e.g. standard deviation) or associated estimates of uncertainty (e.g. confidence intervals)
☐	☒	For null hypothesis testing, the test statistic (e.g. *F*, *t*, *r*) with confidence intervals, effect sizes, degrees of freedom and *P* value noted<br>*Give P values as exact values whenever suitable.*
☒	☐	For Bayesian analysis, information on the choice of priors and Markov chain Monte Carlo settings
☒	☐	For hierarchical and complex designs, identification of the appropriate level for tests and full reporting of outcomes
☐	☒	Estimates of effect sizes (e.g. Cohen's *d*, Pearson's *r*), indicating how they were calculated

Our web collection on statistics for biologists contains articles on many of the points above.

Software and code

Policy information about availability of computer code

Data collection	SosciSurvey was used to collect demographic data and various questionnaires. Else, all data collection (physiological stress data, subjective stress data) was collected without the usage of software.
Data analysis	Analysis scripts and data are publicly accessible at https://osf.io/qg9yc/. Analyses were performed in R 4.2.2 (R Core Team, 2022). OpenFace 2.2.0 was used to analyze AU activity from the participant videos.

For manuscripts utilizing custom algorithms or software that are central to the research but not yet described in published literature, software must be made available to editors and reviewers. We strongly encourage code deposition in a community repository (e.g. GitHub). See the Nature Portfolio guidelines for submitting code & software for further information.

Data

Policy information about availability of data

All manuscripts must include a data availability statement. This statement should provide the following information, where applicable:

- Accession codes, unique identifiers, or web links for publicly available datasets
- A description of any restrictions on data availability
- For clinical datasets or third party data, please ensure that the statement adheres to our policy

Analysis scripts and data are publicly accessible at https://osf.io/qg9yc/.

Human research participants

Policy information about studies involving human research participants and Sex and Gender in Research.

Reporting on sex and gender

Data on sex was collected in terms of self-report. No data on gender was collected.

Our sample consisted of N = 77 parents (41 women, 36 men, age M = 44.9, SD = 5.09, age range = 35-62) accompanied by their adolescent children (40 girls; 37 boys; age M = 14.2, SD = 1.06, age range = 13-16).

Our study was designedd on a cross-sectional balance between parent sex and adolescent sex.

No analyses were perfomed on participant sex. Thus, results are not sex specific.

Population characteristics

Our sample consisted of N = 77 parents (41 women, 36 men, age M = 44.9, SD = 5.09, age range = 35-62) accompanied by their adolescent children (40 girls; 37 boys; age M = 14.2, SD = 1.06, age range = 13-16).

A healthy sample was investigated, meaning that "current diagnosis and treatment categories" does not apply.

Recruitment

Participation for the study was promoted in and around the city of Jena. Before being invited to the laboratory, parents completed a standardized telephone interview assessing inclusion and exclusion criteria. These criteria were chosen to ensure that cortisol measurements were unconfounded and participants could be safely confronted with a psychosocial stress test. Parent-adolescent dyads were excluded if mothers or fathers were strong smokers (> 10 cigarettes a day), reported recreational drug consumption, were unwilling to abstain from alcohol intake for at least a week, had a BMI <18.5 or >30, had previously completed a standardized laboratory stressor, or if either parents or adolescents reported dyslexia or non-fluency in German.
In terms of current physiological and psychological health, dyads were excluded in case of significant health problems, recent stressful life events such as separation or death of a partner/parent, ongoing psychotherapy, diagnosed mental disorders in the last 2 years, or usage of medication affecting HPA axis activity (e.g. steroids). Daughters were excluded from the study if they reported usage of hormonal contraceptives or were pregnant. At the beginning of data collection, mothers were also excluded if they reported usage of hormonal contraceptives, were pregnant or in menopause. However, these restrictions were dropped due to sampling difficulties, which led to the inclusion of 12 mothers (29.3 % of mothers) not on a natural cycle. In detail, four mothers reported using a hormonal coil (9.76 %), four mothers reported being in menopause (9.76 %) and four mothers reported using oral contraceptives (9.76 %).

Ethics oversight

The study was approved by the Research Ethics Board of Jena University Hospital (ethic number: 2019-1578). Parents and children provided written informed consent for both themselves and their children and were financially compensated for their time and effort. Participants were informed that they could withdraw from the study at any point in time.

Note that full information on the approval of the study protocol must also be provided in the manuscript.

Field-specific reporting

Please select the one below that is the best fit for your research. If you are not sure, read the appropriate sections before making your selection.

☐ Life sciences ☒ Behavioural & social sciences ☐ Ecological, evolutionary & environmental sciences

For a reference copy of the document with all sections, see nature.com/documents/nr-reporting-summary-flat.pdf

Behavioural & social sciences study design

All studies must disclose on these points even when the disclosure is negative.

Study description

Cross-sectional study

Research sample

Our sample consisted of N = 77 parents (41 women, 36 men, age M = 44.9, SD = 5.09, age range = 35-62) accompanied by their adolescent children (40 girls; 37 boys; age M = 14.2, SD = 1.06, age range = 13-16).

This sample is representative for healthy parents and adolescents in Germany.

Sampling strategy

Dyads were sampled via snowball and convenience procedures, given that investigating parent-child dyads in a three-hour laboratory study with various exclusion criteria was very difficult. Especially during COVID.

A-priori power analysis. For a one-sided t-test of a single regression coefficient in our multiple linear regression models with six predictors each (parent-AUCi, mimicry, an interaction, puberty status, hormonal status and dyad type), expecting an effect size of at least f^2 = .10 (Blasberg et al., 2023), at an alpha level of 0.05 and a power level of .80, a minimum sample size of N = 64 dyads was

required.
Sensitivity power analysis.
The final cortisol and STAI models included N = 70 dyads. A sensitivity power analysis revealed the sample size has 80% power to detect an effect size of at least $f^2 = .09$.
The final heart rate and HRV models included N = 37 dyads. A sensitivity power analysis revealed the sample size has 80% power to detect an effect size of at least $f^2 = .17$.
The final empathic concern model included N = 71 dyads. A sensitivity power analysis revealed the sample size has 80% power to detect an effect size of at least $f^2 = .09$.
The final personal distress model included N = 69 dyads. A sensitivity power analysis revealed the sample size has 80% power to detect an effect size of at least $f^2 = .09$.

Data collection

Physiological and subjective-psychological stress markers was assessed repeatedly (N = 9) and simultaneously in both parents and their children.

D1. Saliva Cortisol: Collected via Salivettes (Sarstedt, Nümbrecht, Germany).
D2. Heart-Rate: Continous ECG (from -40 to + 40 min), using a Zephyr Bioharness 3 chest belt (Zephyr Technology, Annapolis, Maryland, USA).
D3. Heart-Rate Variability: Continous ECG (from -40 to + 40 min) using a Zephyr Bioharness 3 chest belt (Zephyr Technology, Annapolis, Maryland, USA).
D4. Subjective Stress: Self-report, State-Trait anxiety inventory (STAI; Spielberger 1981).

Only in children:
I1. State Empathy: Self-report, Empathic Response Scale (ERS; Batson et al., 1997).
I2. Puberty State: Self-report, German Version of the Pubertal Development Scale (PDS; Petersen et al., 1988).

Both in parents and children:
I3. Shared Action Unit (AU) activity derived from videos: Both parents and children will be filmed during the TSST. AU coocurrance and AU intensity in both dyad members will be extracted using OpenFace (Baltrušaitis et al., 2018) or a tool with comparable functionality. Shared AU activity will be estimated using windowed cross-lagged correlation analysis (WCLC).
I4. Demographic variables: Age, sex, hormonal status (women), body mass index via SosciSurvey

Timing

Data collection began in June 2022 and ended in March 2024

Data exclusions

No data was excluded.

Non-participation

None of the participants dropped out of the study. The majority of applicants (about 70%) were excluded from study participation due to our strict inclusion and exclusion criteria given the confrontation with a potent psychosocial stressor.

Randomization

Participants were not randomly sampled into groups.

Reporting for specific materials, systems and methods

We require information from authors about some types of materials, experimental systems and methods used in many studies. Here, indicate whether each material, system or method listed is relevant to your study. If you are not sure if a list item applies to your research, read the appropriate section before selecting a response.

Materials & experimental systems

n/a	Involved in the study	
☒	☐	Antibodies
☒	☐	Eukaryotic cell lines
☒	☐	Palaeontology and archaeology
☒	☐	Animals and other organisms
☒	☐	Clinical data
☒	☐	Dual use research of concern

Methods

n/a	Involved in the study	
☒	☐	ChIP-seq
☒	☐	Flow cytometry
☒	☐	MRI-based neuroimaging
